# Immunotherapy-Associated Atherosclerosis: A Comprehensive Review of Recent Findings and Implications for Future Research

**DOI:** 10.1007/s11936-023-01024-0

**Published:** 2023-12-15

**Authors:** Antonia Chan, Stefan Torelli, Evaline Cheng, Ryan Batchelder, Sarah Waliany, Joel Neal, Ronald Witteles, Patricia Nguyen, Paul Cheng, Han Zhu

**Affiliations:** 1 Department of Medicine, Stanford University School of Medicine, Stanford, CA USA; 2 Department of Medicine, Division of Cardiovascular Medicine, Stanford University School of Medicine, Stanford, CA USA; 3 Department of Medicine, Division of Oncology, Stanford Cancer Institute, Stanford University School of Medicine, Stanford, CA USA; 4 Stanford Cardiovascular Institute and Department of Medicine, Stanford University, 240 Pasteur Drive, Rm 3500, Biomedical Innovations Building, Stanford, CA 94304 USA

**Keywords:** Cardio-oncology, Atherosclerosis, Immunotherapy

## Abstract

**Purpose of the Review:**

Even as immune checkpoint inhibitors (ICIs) have transformed the lifespan of many patients, they may also trigger acceleration of long-term cardiovascular disease. Our review aims to examine the current landscape of research on ICI-mediated atherosclerosis and address key questions regarding its pathogenesis and impact on patient management.

**Recent Findings:**

Preclinical mouse models suggest that T cell dysregulation and proatherogenic cytokine production are key contributors to plaque development after checkpoint inhibition. Clinical data also highlight the significant burden of atherosclerotic cardiovascular disease (ASCVD) in patients on immunotherapy, although the value of proactively preventing and treating ASCVD in this population remains an open area of inquiry. Current treatment options include dietary/lifestyle modification and traditional medications to manage hypertension, hyperlipidemia, and diabetes risk factors; no current targeted therapies exist.

**Summary:**

Early identification of high-risk patients is crucial for effective preventive strategies and timely intervention. Future research should focus on refining screening tools, elucidating targetable mechanisms driving ICI atherosclerosis, and evaluating long-term cardiovascular outcomes in cancer survivors who received immunotherapy. Moreover, close collaboration between oncologists and cardiologists is essential to optimize patient outcomes.

## Opinion statement

The current treatment options for ICI-mediated atherosclerosis require extensive further investigation given the comparatively recent awareness of this phenomena. Targeted therapies are limited and management is still heavily informed by evidence for traditional age and lifestyle-related ASCVD, although preclinical evidence suggests that immune dysregulation plays a much more prominent role in ICI-mediated atherosclerosis. One key component is monitoring and optimizing baseline cardiovascular risk factors such as hypertension, hyperlipidemia, and diabetes, while engaging in lifestyle modifications such as smoking cessation, a low-fat diet, and regular exercise as tolerated. Radiographic imaging remains an underutilized tool for assessing baseline plaque burden, identifying future lesional areas, and predicting the risk of severe cardiovascular events in patients on ICIs. However, further research is needed on how to best utilize and hybridize standard-of-care CT/FDG-PET scans for oncology patients with cardiac imaging modalities that track progression of coronary artery calcium and vascular wall inflammation. Therapeutically, standard medical therapy including statins, ezetimibe, PCSK-9 inhibitors, and antiplatelet agents can be employed as tolerated. There is some evidence that statins in particular enhance the effectiveness of ICI while attenuating plaque progression; however, clinicians should be aware of the potential increased risk for myopathy. Nonspecific immunomodulatory agents such as steroids are not recommended for prevention or suppression of ICI-induced plaque development, as they may worsen atherosclerosis. While targeted therapies that address the underlying immune-mediated mechanisms are still being developed, optimal management of ICI atherosclerosis should emphasize recognition of immunotherapy as a major ASCVD risk factor, early risk stratification/optimization, and a multidisciplinary approach involving both oncologists and cardiologists to determine appropriate screening and medical management.

## Introduction

Immune checkpoint inhibitors (ICIs) have dramatically changed cancer therapy over the last decade, allowing patients with many malignancies to live longer with better quality of life, particularly those with melanoma and non-small cell lung cancer (NSCLC). Over thirty percent of all cancer patients are now eligible for ICI therapy, while up to 70% of ICI-treated patients may go on to develop an immune-related adverse event (IRAE) [1]. By reducing immune tolerance of malignant cells, ICIs inherently carry the risk of off-target T-cell autoreactivity in other organs. Much research on ICI cardiotoxicity has focused on lymphocytic myocarditis, a rare IRAE with a high mortality rate [2]. A growing body of basic science, translational, and clinical evidence now suggests that myocarditis may be the tip of the iceberg in terms of cardiac IRAEs, with ICI-induced acceleration of atherosclerotic cardiovascular disease (ASCVD) contributing significantly to vascular toxicity in the long term.

Disentangling the relationship between cardiovascular disease, cancer, and cancer treatment is complicated by shared risk factors such as older age, comorbid conditions (e.g., hypertension, diabetes, and dyslipidemia) and a chronic inflammatory state. Initially viewed as a bland lipid storage disorder, atherosclerosis is now thought to be exacerbated by an inflammatory milieu. Observational and prospective epidemiological studies have demonstrated that higher levels of IL-6, TNF-α, fibrinogen, and CRP are associated with increased cardiovascular risk [3–7]. Atherosclerosis-specific self-antigens include LDL, oxidized LDL (oxLDL), heat shock protein, and ApoB [8–11]. In both human and mouse models, endothelial expression of vascular cell adhesion molecules and T-cell chemoattractants is upregulated within early atheromas, which in turn facilitate binding and migration of macrophages and T-lymphocytes at these sites [12–15]. Once inside the arterial wall, immune cells promote evolution of plaques and contribute towards their acute thrombotic complications: macrophages ingest lipids to become foam cells, while T cells secrete inflammatory cytokines that further stimulate macrophages and smooth muscle cells (SMCs) that make up plaque. These cytokines—particularly IFN-γ—ultimately contribute to risk of acute thromboses through growth and destabilization of plaques and their fibrous cap [16–22]. Systemically circulating acute phase reactants such as fibrinogen and plasminogen may also alter thrombotic risk [19].

Targeting inflammation to reduce ASCVD risk in humans has been previously tested with mixed results. CANTOS (Canakinumab Anti-Inflammatory Thrombosis Outcomes Study), LoDoCo (Low-Dose Colchicine), and COLCOT (Colchicine Cardiovascular Outcomes Trial) support the theory that inhibition of innate immunity slows the progression of ASCVD [23]. However, the CIRT trial (Cardiovascular Inflammation Reduction Trial) did not find that low-dose methotrexate reduced cardiovascular events or levels of IL-1β, IL-6, or CRP. CANTOS also showed only modestly (− 15%) reduced cardiovascular events in post-myocardial infarction patients [24]. These trials highlight how immunomodulatory therapies for atherosclerosis must target specific points of immune dysregulation, which are still not fully understood.

The majority of cancers are associated with increased systemic inflammation, which is thought to contribute to the significantly increased risk of thrombosis in patients with active cancer. These risks are heightened by radiation therapy, which accelerates plaque development and calcification, as well as certain tyrosine kinase inhibitors which have direct vascular toxicities [25–30]. Certain malignancies, such as pancreatic or advanced genitourinary cancer, also carry an independently high thrombogenic risk [31]. More recently, accelerated ASCVD has emerged as an important frontier in understanding ICI cardiotoxicity beyond myocarditis [23].

Here, we review the latest clinical and preclinical literature on the epidemiology and mechanisms of ICI atherosclerosis. With heart disease a leading cause of death among cancer survivors, and ICIs increasingly used as adjuvant treatment, understanding the causative pathways behind ICI-mediated ASCVD can help clinicians thread the needle between maximizing cancer treatment response while mitigating serious potential long-term cardiac toxicities.

## Clinical evidence supporting ICI-mediated atherosclerosis

Several recent studies suggest an association between ICI initiation and rates of subsequent atherosclerotic cardiovascular events [23]. In one large matched cohort study involving 2842 patients who started immunotherapy and 2842 controls matched by age, the 2-year risk of myocardial infarction (*n* = 37, 1.30%), coronary revascularization (*n* = 22, 0.77%), or ischemic stroke (*n* = 35, 1.23%) after starting an ICI was threefold higher, with > threefold higher rate of plaque progression on imaging [32•]. Interestingly, this association was attenuated by concomitant use of statins or corticosteroids, though interpretation of these results is limited by potential confounding by indication. Another study by Bar et al. reported that among 1215 cancer patients receiving ICI therapy, 31 (2.6%) developed acute vascular events and 8 (0.66%) developed myocardial infarction within 6 months of treatment initiation [33].

Smaller scale studies and prior meta- or pooled-analyses differ in their conclusions on the burden of ICI-mediated ASCVD. In patients with non-small cell lung cancer, prior studies have shown no definitive increase in cardiovascular or venous/arterial events relative to traditional cytotoxic therapy [33, 34]. One meta-analysis of treatment-related deaths in FDA-approved PD1/PDL1 clinical trials (*n* = 82) found a 9.8% incidence of deaths attributable to cardiovascular events, although incidence of myocardial infarction (*n* = 1, 1.2%) and acute coronary syndrome (*n* = 1, 1.2%) was low [35]. Conversely, an FDA pooled analysis of patients on ICI therapy (*n* = 21,644) suggested up to a 35% (95% CI: 0.76 to 2.4) increase in isolated coronary ischemia over 6 months for patients on an ICI versus traditional cytotoxic therapy [34].

Radiographically, patients with lung cancer and melanoma do appear to develop more unstable plaque phenotypes after immunotherapy. Non-calcified plaques are associated with greater cardiovascular and stroke risk than calcified plaques, while carotid plaque inflammation has been linked to less calcification on imaging [36]. One case–control study (40 cases with ICI and 20 controls without ICI) by Drobni et al. found that ICI use in lung cancer was associated with a significantly higher progression rate of noncalcified plaque volume (11.2% vs. 1.6% per year, *p* = 0.001). Patients who did not receive immunotherapy showed greater progression in calcified plaque volume (25% vs. 2% per year, *p* = 0.017) [37]. Similarly, in a cohort of 35 patients with melanoma and pre-existing aortic calcification, post-ICI plaque composition showed increased non-calcified plaque volume, which suggests a more vulnerable, rupture-prone state [36]. While existing studies often include a low proportion of patients on combination ICIs, making it difficult to assess if combination immunotherapy enhances ICI-mediated atherosclerosis, Drobni et al. reported that patients on combination ICIs exhibited greater plaque progression, suggesting that multiple checkpoint blockades have an additive effect on vascular inflammation [32•].

Atherosclerosis is a gradual process in which the disease burden progresses over many years or even decades. Improved oncologic outcomes have extended survival times, meriting closer attention to the potential health complications of longer term cancer survivorship. Follow-up evaluation of patients over a greater time window is needed to fully understand the chronic impact of ICI therapy on ASCVD and how it interacts with other cardiovascular risk factors such as age, hypertension, and diabetes.

## Preclinical models for ICI atherosclerosis

The role of T cells in atherosclerosis has come to attention in recent years, particularly as innovative single-cell analyses in human and mouse models shed new light on the immune diversity of plaques beyond macrophages [38–43]. T cells are the predominant lymphocyte type in plaque, but murine models have reported both pro- and anti-atherogenic functions of different T cell subsets [8]. In mice, CD4+ T helper 1 (Th1) cells appear to be pro-atherogenic in large part due to production of IFN-γ and TNF-α [44–49]. The role of other Th subsets, CD8+ T cells, and Tregs remains equivocal, potentially due to differences in mice strain, T cell depletion strategy, gene knockout strategy, and study diet [8, 50]. There is also a lack of knowledge around antigen specificity for these T cell subsets, so it cannot be ruled out whether a particular Th subset is pro- or anti-atherogenic depending on the type of antigens or signaling present. For example, while generally thought to be atheroprotective in mice due to secretion of TGF-β and IL-10, Tregs’ initial protective response may shift towards pro-inflammatory as atherosclerosis progresses [51–57]. In humans, there is also not a consistent clinical correlation between blood/plaque Treg level and atherosclerosis [58–61]. Depuydt et al. did find that autoreactive CD4+ T cells may contribute to atherosclerosis in humans [43]. Fernandez et al. further suggest that T cell activation and exhaustion reprogramming play an important role, with exhausted human plaque T cells expressing more PD-1 than their blood counterparts [41•]. This has sparked interest in the potential impact of immunotherapy on plaque T-cell activity.

**Fig. 1:**
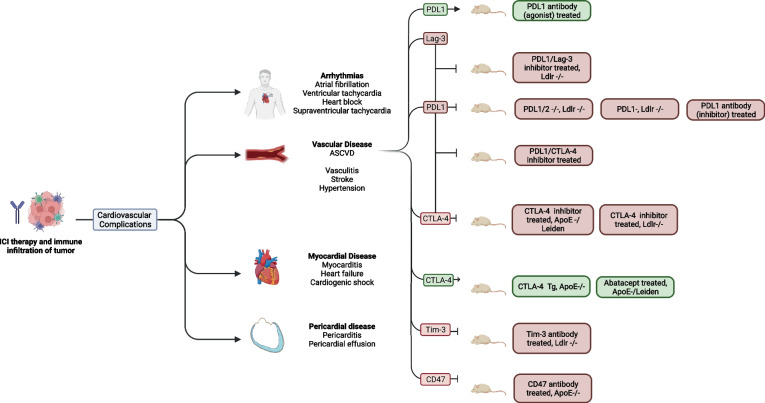
This figure shows the different cardiac toxicities of immunotherapy and the different mouse models that have been used to investigate ICI-associated atherosclerosis. Abbreviations: PDL-1, programmed cell death ligand 1; Lag-3, lymphocyte activation gene 3; *CTLA-4*, cytotoxic T-lymphocyte antigen 4; Tim-3, T-cell immunoglobulin mucin; ApoE, apolipoprotein E; Ldlr, low-density lipoprotein receptor.

By releasing the innate brake on T cell activation, ICIs promote a systemic, T-cell mediated antitumor response. However, disrupting this key regulatory step can also result in many off-target immune reactions. Figure 1 and Table 1 summarize preclinical studies that have used genetic knockout models and co-stimulation/co-inhibition of ICI targets to shed light on the role of immune checkpoints in atherosclerosis.

**Table 1:** Mouse models of immune checkpoint inhibitor induced atherosclerosis

Model	Study	CD4*	CD8*	Treg*	Macrophages*	Plasma TNF-a or IFN-g
Pdl1/2-/-Ldlr-/- mice	Gotsman et al. (2007) [66]	↑	↑		↑	
Pdl1-/-, Ldlr-/-	Bu et al. (2011) [62] Cochain et al. (2014) [63]	↑ ↑	↑ ↑		↑ Unchanged	↑
Pdl-1 antibody (inhibitor) treated	Bu et al. (2011) [62] Zhang et al. (2017) [65]	↑ ↑	↑ ↑		↑	
Pdl-1 antibody (agonist) treated	Grievink et al. (2021) [67]	↓	↓			↓
Pdl-1/Ctla-4 antibody treated	Poels et al. (2020) [75]	↑	↑			
Ctla-4 antibody treated, Apoe Leiden or Apoe-	Ewing et al. (2013) [72] Ma et al. (2013) [73]	Unchanged (splenic) ↓		↓ (splenic)	↓	↓ (splenic)
Ctla-4 antibody treated, Ldlr-/-	Poels et al. (2020) [74]	↑ (splenic)	Unchanged (splenic)	Unchanged (splenic)	↓ (splenic)	
Ctla-4 Tg/Apoe-	Matsumoto et al. (2016) [71]	↓		Unchanged	↓	↓
Pd-1 and Lag-3 antibody treated, Ldlr-/-	Mulholland et al. (2022) [77]	↑	↑			↑
Tim-3 antibody treated, Ldlr-/-	Foks et al. (2013) [163]			↓	↑	
CD47 antibody treated, ApoE -/-	Kojima et al. (2016) [82]				↑	

↑ indicates an increase in cell count or serum level. ↓ indicates a decrease in cell count or serum level

*PDL-1* programmed cell death ligand 1, *Lag-3* lymphocyte activation gene 3, *CTLA-4* cytotoxic T-lymphocyte antigen 4, *Tim-3* T-cell immunoglobulin mucin, *ApoE* apolipoprotein E, *Ldlr*, low-density lipoprotein receptor

*Taken from atherosclerotic lesions unless otherwise specified

The PD-1/PDL-1 pathway was one of the original targets of ICI therapy, but also plays an important role in downregulating T-cell activity. Bu et al. showed that Pd1−/−Ldlr−/− mice had not only worsened atherosclerotic disease but also enhanced infiltration of cytotoxic T cells and macrophages in plaques and arterial walls, as well as elevated levels of serum IFN-γ and TNF-α, suggesting continued immune dysregulation [62]. Similarly, other studies in PD1-deficient mice or mice treated with anti-PD1 antibodies have reported that these mouse model experience worsened plaque burden with significant T cell and macrophage expansion both in atherosclerotic lesions and systemically [63–65]. Bu et al. also reported that Pd-l1/2-/- Ldlr -/- bone marrow chimeric mice had significantly more CD4+ T cells, CD8+ T cells, and macrophages in plaque lesions compared to controls; this difference was most notable for CD8+ T cells [62]. These results are consistent with Gotsman et al.’s prior work, which showed that Pd-l1/2-/- Ldlr-/- mice had more lesions and lesional inflammation [66]. Stimulation of the PD-1 pathway in a drug-treated mouse model appeared to have an opposite effect on atherosclerosis by decreasing levels of monocytes and T cells that secrete IFN-γ in the peripheral blood, while improving biomarkers such as B1 cells, Bregs, and oxLDL IgM levels considered to be atheroprotective [67]. Beyond the role of PD-1/PDL-1 in T cell exhaustion, a PD1-deficient mouse model with selective targeting of myeloid cells suggested that this pathway also acts as an important negative regulator of cholesterol synthesis and uptake [68]. Collectively, these preclinical studies provide robust evidence that inhibiting the PD1/PDL-1 pathway accelerates atherosclerotic plaque progression.

The CTLA-4 pathway plays an important atheroprotective role by competing with CD28 in T cell activation and acting as a co-inhibitor of T cell migration into nonlymphoid tissues such as arterial walls or tumors [69, 70]. CTLA-4 Tg/Apoe-/- mice, which constitutively over-express CTLA-4, have been found to have less plaque formation as well as markedly reduced intralesional and vessel wall accumulation of macrophages and CD4+ T cells [71]. Treatment with abatacept, which inhibits *CD28* mediated T-cell activation, also mitigated the accelerated atherosclerosis and elevated serum levels of IFN-γ seen in ApoE3-Leiden and Apoe -/-mice [72, 73]. Conversely, Poels et al. reported that antibody-mediated inhibition of the CTLA-4 pathway in Ldlr-/- mice induced an activated T cell profile and increased ICAM1 endothelial expression along with increases in aortic plaque area [74].

PD-1/PDL-1 and CTLA4 remain the most common and widely used targets for cancer immunotherapy. Short-term combination immunotherapy led to unfavorable plaque and artery wall profiles in Ldlr-/- mice in the setting of T-cell, not myeloid, driven inflammation: larger necrotic core size, increased intraplaque CD8 T cells, increased CD4 effector/CD8+ T cells in arterial walls, and more endothelial activation as measured by *Icam1* expression [75].

More recently, combination therapy with PD-1 and LAG3 blockade was approved for patients with unresectable or metastatic melanoma [76]. LAG3 is expressed across multiple leukocyte subtypes including T cells. Within T cells, it negatively regulates T cell activation and proliferation and is expressed at higher levels on exhausted T cells [77]. Mulholland et al. used Ldlr-/- mice as a background to test the effect of a Lag3 genetic knockout, as well as treatment with an anti-Lag3 antibody. In both models, there was no change in plaque burden, but recruitment of T cells to plaque lesions and increased levels of T cell activation/cytokine production were observed. They also noted an additive effect of dual LAG3/Pd-1 blockade on T cell activation [77].

TIM-3, a co-inhibitory immune checkpoint expressed on CD8 T cells, may also be a negative regulator of atherosclerosis. Foks et al. report that in Ldlr- mice fed a lipid-rich diet, TIM-3 antibody blockade increased macrophage content and decreased Treg cells in atherosclerotic plaque [23]. This complements findings in human subjects treated with dual inhibition of PD-1 and TIM-3, who had increased pro-inflammatory cytokine levels (IFN-g and TNF-a) compared to singular blockade of either immune checkpoint [78].

Conversely, magrolimab, an anti-CD47 monoclonal antibody that is part of the macrophage checkpoint inhibitor class, appears to exert anti-atherogenic effects in recent human and animal studies. Macrophages play an important role in programmed cell removal of apoptotic or diseased cellular debris through a process called efferocytosis, leading to interest in magrolimab’s potential to induce tumor cell phagocytosis [79]. A retrospective study of 9 lymphoma patients treated with magrolimab and rituximab also showed suppression of vascular inflammation on FDG-PET, although it did not assess how plaque composition or efferocytosis rates were directly modified by magrolimab [80]. The pro-atherogenic effects of CD47 are thought to be from nonspecific downregulation of efferocytosis, as a key feature of plaque is the formation of a necrotic core of apoptotic lipid-laden cells [81]. Kojima et al. recently reported that treatment with CD47 antibodies attenuated atherosclerosis across multiple mouse models, consistent with their findings that CD47 is also upregulated in atherogenesis and localizes intensely to plaque necrotic core [82].

Preclinical models of atherosclerosis in other autoinflammatory conditions offer further insight into the cardiovascular sequelae of dysregulated adaptive immune activity that ICIs may mimic. Rheumatoid arthritis (RA) and systemic lupus erythematosus (SLE) have well-established associations with the development of cardiovascular disease. MRL-Faslpr mice, which develop an SLE-like phenotype, are at higher risk of atherosclerotic disease and vascular damage, potentially driven by leukocyte infiltration and over-expression of inflammatory cytokines such as IL-6 and TNF-α [83, 84]. While MRL-Pdcd1-/- mice have been found to be predisposed to lymphocytic myocarditis, there are no current studies which examine atherosclerosis in MRL-Pdcd1-/- mice [85]. The B6.SLE mouse model also leads to a lupus phenotype involving T cell activation. Transfusion of isolated CD4+ T cells from this model into Ldlr-/-, Rag-/- mice (lacking functional B and T cells) dramatically accelerated atherosclerosis without causing lupus in the recipient [86].

In summary, recent preclinical models of atherosclerosis suggest that immune checkpoint pathways play a vital role in down-regulating T cell activation and migration. Further research is required to understand whether T cell activation and plaque progression worsen with a “dual-hit” of checkpoint blockade compared to ICI monotherapy, as risk of checkpoint myocarditis has been shown to. Finally, most studies utilized complete genetic knockout/chimeric mice or administration of an antibody drug not used for treatment of human subjects. This may limit their generalizability due to potential treatment effects on unexamined cell subtypes. For example, the role of myeloid versus T cell PD-1 blockade is still not well-understood. Strauss et al. found that targeted PD1 deficiency in myeloid-specific (Pd1 f/fLysMcre) mouse cells appeared to decrease tumor growth more than T cell specific (Pd1 f/fCD4cre) targeting [68]. At the same time, the myeloid-targeted mice had significantly elevated cholesterol levels, which may be consistent with clinical research showing baseline hypercholesterolemia and obesity’s association with improved survival in human patients treated with ICIs [87–89].

## Proposed mechanisms for ICI atherosclerosis

No direct mechanistic studies in humans have been performed to evaluate accelerated atherosclerosis in the setting of ICI therapy. However, extrapolating from mouse models for atherosclerosis and ICI myocarditis provides insight into several plausible pathways for immune checkpoint blockade to cause plaque progression. Single-cell sequencing and mass cytometry showed that T-cells are active contributors in human and mice atherosclerotic lesions [51, 90]. These plaques exhibit significant heterogeneity in CD4+ and CD8+ T-cell phenotypes, ranging from highly active T cells expressing inflammatory molecules like granzymes and cytokines, to exhausted T cells characterized by high PD1 expression. Interestingly, in late-stage atherosclerosis, three major T cell subsets appear to undergo clonal expansion, indicating that generation of autoreactive CD4, CD8, and Treg cells may play an important role in ASCVD progression [91]. Conversely, inhibition of costimulatory T cell activation in mice through targeting of CD40/CD40L and B7 led to reduced plaque size and formation [92–94]. However, while the importance of T cell immunity in atherosclerosis is well established, the exact site of T-cell activity-associated atherosclerosis remains unclear.

One theory is that checkpoint inhibition of PD1 could activate T cells and decrease peripheral tolerance within intimal plaques [38, 95], leading to increased risk of plaque rupture and MACE. This has not been experimentally verified and may not be generalizable to explain higher rates of ASCVD seen in patients undergoing non-PD-1/PDL-1 treatments. Furthermore, in patients with basal or squamous cell carcinoma treated by PD-1 blockade, pre-existing tumor-specific T cells appeared to have limited reactivation capacity, and response to therapy may have resulted instead from constant attraction of new T cells [96].

Another proposed mechanism for ICI atherosclerosis is the unleashing of antigen-experienced T cells to recognize atherosclerosis-specific autoantigens. Recent mouse model studies of myocarditis have confirmed the presence of antigen-specific effector-like cytotoxic CD8 T cells which recognize cardiac autoantigens such as alpha-myosin [97–99]. It is still unclear whether these findings can be extrapolated to ICI atherosclerosis, and if the latter is indeed an antigen-driven process, what the identities of these specific antigens might be [91]. Single-cell RNA analysis of human coronary atherosclerotic plaque has demonstrated clonal expansion of primarily antigen-experienced T-cells, highlighting one potential pathway for self-reactive epitopes to accelerate atherosclerosis by interacting with smooth muscle cells and macrophages in the plaque microenvironment [100].

Recent research in Apoe-/- mice with advanced atherosclerosis found patterns of deteriorating peripheral T-cell tolerance, most notably in plaques followed by artery tertiary lymph node organs [91]. The large number of immune checkpoint pathways beyond PD-1/PD-L1 and CTLA4 has not been as closely examined for their role in modulating peripheral T cell tolerance in the setting of cancer and atherosclerosis. Further research is required to determine how much T-cell recruitment versus T cell reactivation drives the accumulation and clonal expansion of various T cell subsets within ICI atherosclerosis.

Functional cross-talk between T cells and macrophages also deserves further investigation in both the plaque and solid tumor immune microenvironments. Lipid-laden macrophages, or foam cells, directly contribute to plaque volume and necrotic core formation. After infiltrating vessel walls, foam cells are thought to enhance activation of the inflammasome and CD4 T cells, particularly Th1 cells, which in turn secrete proatherogenic IFN-γ and TNF-α. [63, 101] Interferons are particularly important regulators of atherosclerosis due to their role in enhancing endothelial activation, regulation of apoptosis, and foam cell formation [102]. It will be important for clinicians to understand how ICI-mediated systemic T-cell activation impacts CD4-monocyte interactions within plaque, whether foam cells in particular retain some degree of monocyte antigen-presenting capabilities, and how T cells may play a reciprocal role in ASCVD-related monocyte function. For example, while CD8 T cells’ contribution to atherosclerosis is less clear, Cochain et al. demonstrated in a Ldlr-/- mouse model that CD8+ T cell activity promoted atherosclerosis through modulation of monopoiesis and serum monocyte levels [103, 104]. Similarly, other murine models in PD1-deficient mice show a shift from CD4+ to CD8+ T cells within vessels affected by plaque, while CD8 T-cell depletion in Apoe- and Ldlr-/- mice reduced atherosclerosis [62, 66]. This was hypothesized to be due to CD8 T cells’ cytotoxic function in macrophage cell death and necrotic core formation within atherosclerotic plaques. ScRNA-sequencing profiles also suggest that dysfunctional CD8 T cell tolerance in mouse plaques is shared by human coronary/carotid arterial plaques [91].

The role of Tregs in ICI atherosclerosis remains an open question. Tregs secrete TGF-b and IL-10 which encourage an anti-inflammatory macrophage phenotype, while constitutively expressing CTLA-4 and PD-1/PDL-1 [105–107]. Increased number of Treg cells is associated with smaller human plaque size and improved plaque stability [107]. In the context of atherosclerosis, however, Treg cells may switch to exTreg cells through Treg/Th17 conversion in plaques [91]. One PD1-/Ldlr- mouse model found that PD1 knockout stimulated the Foxp3 + Treg response systemically and in atherosclerotic vessels, but overall, a pro-atherogenic CD4+ T cell response dominated [63]. Although studies have shown differing conclusions on PD-1/PDL-1’s regulation of Treg function, Tregs appear to play an overall negative regulatory role in T cell activation that may counteract the pro-inflammatory cascade triggered by blockade of CTLA-4 or PD1/PDL1 [108, 109]. Current evidence on how Treg function is affected by other checkpoints targeted in ICI therapy, such as LAG-3 is sparse. Data from genetic knockout mice suggest that LAG-3 promotes autoimmunity by limiting Treg cell proliferation/function [110].

Beyond primary ICI atherosclerosis, immunotherapy may exacerbate atherosclerosis through alternative IRAEs such as vasculitis. Among the systemic vasculitides, accelerated atherosclerosis has been clearly noted in Takayasu arteritis and ANCA-associated vasculitis (AAV) [111]. Systematic reviews and pharmacovigilance studies have reported that anti-PD-1 and CTLA-4 directed therapy have been associated with large-vessel vasculitis and vasculitis of the nervous system [112–114]. Interestingly, a humanized mouse model of GCA found that treatment with anti-PD-1 antibodies led to arterial infiltration with T cells and macrophages even from healthy subjects and development of fulminant arteritis [65]. Native AAV has been linked to single nucleotide polymorphisms in the CTLA-4 gene, highlighting one possible mechanism through which CTLA-4 inhibition may also lead to vasculitis [115].

Collectively, these studies point to immune checkpoint pathways as important negative regulators of T-cell activation, function, and recruitment. Long-term, ICI-mediated ASCVD may result from disrupting these mechanisms’ role in artery walls and plaques.

## Treatment options

### Prevention and monitoring

Several recently completed trials suggest that the adjuvant/neoadjuvant use of immunotherapy leads to additional improvement in survival rates for patients with resectable disease, particularly those with NSCLC or melanoma [116–121], [122–126]. As immunotherapy shifts towards curative or long-term adjuvant use for some patients, it becomes increasingly important that clinicians recognize and implement ICI-mediated ASCVD screening as part of routine cancer treatment. This may require coordination between primary care providers, oncologists, and potentially cardiologists to screen for and manage modifiable risk factors such as cholesterol levels, diabetes mellitus, and hypertension.

Cross-specialty collaboration is also needed to determine the optimal use and timing of imaging techniques. Plaque progression is a strong predictor of future cardiovascular events. Drobni et al. found that among 40 patients with melanoma, there was a greater progression rate of thoracic plaque burden (2.1% per year to 6.7% per year, *p* = 0 0.02) on CT after initiation of ICI [32•]. In a smaller cohort of 11 patients with pre-existing ASCVD on nivolumab in Italy who received contrast-enhanced CT scans at baseline and a minimum of 8 weeks, 63.6% had no significant changes and 27.3% experienced significant improvement in plaque burden; only one patient showed modest worsening of atherosclerotic lesions, and one patient demonstrated repeated, dramatic resolution of plaques on treatment with PD1/PDL1 inhibitors [127, 128]. Interestingly, all cases with significant improvement in plaque burden also reported grade > 2 IRAEs [127]. The imaging results of FDG PET-CT studies assessing systemic inflammation and plaque burden after ICI in small-scale cohorts of melanoma patients and mouse models have also been inconclusive, although Calabretta et al. do suggest that immunotherapy may trigger low-grade vascular wall inflammation primarily affecting the earlier, more vulnerable non-calcified coronary plaques, which could increase risk of future rupture [129, 130].

Going forward, it is important for clinicians to recognize the benefits and limitations of standard oncologic imaging approaches as a means of screening for and monitoring ASCVD in patients on immunotherapy [131]. Incidental coronary artery calcium (CAC) seen on non-gated CT imaging has been found to be predictive of major adverse cardiac events, particularly when the CAC score was over 100. However, prospective randomized trials examining the impact of non-gated CAC on ASCVD outcomes are needed [132, 133]. Future studies incorporating the use of coronary CT angiography to evaluate for ICI-mediated atherosclerosis are also needed. Novel imaging approaches such as immune-PET tracers are currently uncommon in clinical practice, but have proven valuable in human and mouse models for detecting vulnerable atherosclerotic lesions generally [134–137].

### Pharmacologic therapy

Treatment strategies for ICI-mediated ASCVD remain in the exploratory phase. Statins, ezetimibe, fibrates, and PCSK9 inhibitors are safe, effective, and well-established treatments for atherosclerosis. However, their impact in the setting of ICIs and cancer requires further exploration to fully characterize risks and benefits to this complex patient population.

### Statins

Despite hyperlipidemia control’s well-known role in ASCVD risk reduction, prospective randomized control trials have not yet evaluated the efficacy of statins from both an onco- and atheroprotective perspective during ICI therapy. In a recent retrospective study, Drobni et al. showed that statin or aspirin use by patients receiving ICI therapy was not associated with differences in a composite outcome of cardiovascular events, but statins were associated with significantly slower annual rate of progression in both total and noncalcified plaque volume in an imaging substudy of 40 patients [32•]. Because patients on statins were more likely to have ASCVD risk factors, it is difficult to interpret Drobni et al.’s findings in isolation. Interestingly, several recent studies report that concomitant use of common cardiovascular medications such as statins or aspirin also improves ICI activity, with enhanced activity of cytotoxic CD8 T cells and reduction of pro-inflammatory cytokines as possible mechanisms [138–140]. Adjuvant statin use during ICI therapy may carry important risks however. Drobni et al. subsequently conducted another retrospective analysis that suggested a > twofold risk of inflammatory or non-inflammatory skeletal myopathy in such cases, with a 2.5-fold higher risk of inflammatory myopathy, although there was not an increased risk of transaminitis [141].

### PCSK-9 inhibitors

PCSK9 inhibitors are a newer class of monoclonal antibodies (and RNAi) used to treat patients at high risk for ASCVD refractory to statins. Similar to statins, recent studies have found that adjuvant PCSK9 use with ICIs, leading to enhanced intratumoral cytotoxic T cell infiltration, antigen presentation, and expression of co-inhibitory checkpoint molecules [142–144].

### Steroids

In retrospective studies, steroid use during ICI therapy has been associated with a lower annual rate of plaque progression (3.5% vs. 6.9%, *p* = 0.04); however, there is a lack of prospective data supporting this association [32•]. Several confounders may still be present including study matching by cancer type, degree of pre-existing ASCVD, and length of steroid or ICI therapy. More importantly, chronic glucocorticoid excess has been associated with worse cardiovascular outcomes, most strikingly in patients with Cushing’s and metabolic syndrome, but also in those with autoimmune conditions requiring exogenous steroids such as lupus or rheumatoid arthritis [145–149]. Inflammation from autoimmune disease itself likely contributes to ASCVD. However, glucocorticoids have non-specific immunosuppressive effects, while also carrying their own increased risk of long-term atherosclerotic complications. Several studies have reported that even low levels of steroids confer an increased risk of ASCVD in these patients when used chronically [148–150]. From an oncologic standpoint, some studies raise the concern that steroids may blunt ICI treatment response, though this is not entirely clear [151–156]. Overall, steroids are not recommended for prevention or treatment of ICI-mediated ASCVD, and more research on targeted steroid-sparing therapies is needed.

### Emerging therapies

Just as ICIs have transformed oncology, they have also changed clinical considerations for patients who experience cardiotoxic IRAEs. While existing literature suggests that ICIs cause a net acceleration in atherosclerosis, we lack a complete mechanistic understanding of their effects on plaque formation and progression. Induction of a pro-inflammatory, T cell-rich environment likely plays an important role. However, ICIs have heterogeneous effects on immune activation, some of which may even be atheroprotective, and newer checkpoint targets such as TIM3 inhibitors remain less studied. Although active research is ongoing into focused immune targets behind plaque formation in ICI-associated atherosclerosis, specific molecular and cell-based therapies remain needed and lacking.

Sex-related differences in ICI cardiotoxicity remain one knowledge gap with implications for ICI atherosclerosis risk stratification. Both clinical and mouse studies suggest that females may be at higher risk of ICI myocarditis, but the literature on sex disparities in ICI atherosclerosis is more equivocal, limited by small sample sizes or borderline significance [32, 33, 157, 158]. In the general population, sex differences in cardiovascular biomarkers (e.g. adipokines, inflammatory markers, fibrosis, and metalloproteinase inhibitors) have been found and were most pronounced between pre-menopausal women versus men [159]. There is also a well-established sex-based dimorphism in risk of autoimmune disease and IRAEs that is increased for women [160–162]. This highlights the complex interplay between several cardiovascular risk factors that may help determine individuals’ risk for ICI atherosclerosis and response to standard therapy.

Moving forward, eliciting the interaction of genetic, hormonal, and immune-related factors in ICI atherosclerosis is critical to preserving cardiovascular health among cancer patients who receive immunotherapy. Expanding our knowledge of these pathways will allow for the basic and translational insights needed to develop novel preventive and therapeutic strategies. For this complex patient population, developing more precise ASCVD risk stratification and targeted therapies while defining current imaging and pharmaceutical best practices will require close collaboration between cardio-oncologists, oncologists, and basic/clinical/translational researchers.

## Conclusion

In addition to their remarkable success in treating many malignancies, ICIs have been linked to increased risk of ASCVD, one of the most common chronic conditions with an underlying inflammatory component. As immunotherapy moves into the curative/adjuvant setting, advanced cancer survivorship is also expected to grow. This population of patients who require ongoing, potentially lifetime, use of ICI therapy to control their advanced or metastatic disease carry unknown cardiovascular risks that can significantly impact mortality beyond their cancer diagnosis. It is essential for all clinicians who treat cancer patients longitudinally to recognize ASCVD as a potential immune-related complication that would benefit from close monitoring and control of risk factors such as hypertension, hyperlipidemia, and diabetes mellitus, especially in those with a prior ASCVD history or risk factors.

The precise mechanisms underlying the pathogenesis of ICI atherosclerosis remain in need of further preclinical and clinical study. Several mouse models of ICI atherosclerosis suggest that checkpoint inhibition disrupts negative regulation of T-cell activity and serum levels of proatherogenic cytokines in particular. Still, it remains necessary to distinguish the extent of T-cell reactivation versus T-cell recruitment during this process, as well as whether Treg function leads to a net atherogenic or atheroprotective effect in the setting of ICI use. Finally, cross-talk between CD4/CD8 T cells and myeloid populations such as macrophages/monocytes also appear to play an important role in plaque development, although the precise impact of immunotherapy on their activity remains under-explored.

Further characterization of the mechanisms underlying plaque progression is needed to determine appropriate timing of atherosclerotic imaging as well as medical interventions. Existing observational studies have limited follow-up timelines and likely do not capture the full extent of the association between ICI use and lifetime ASCVD risk or other adverse cardiovascular events. Longer-term trials using larger cohort sizes are also needed to understand how standard medical management of ASCVD risk factors affects cancer patients in heterogeneous ways. Our review underscores the importance of further preclinical and clinical investigation into this growing patient population to explore targeted therapeutic targets.
